# Teledentistry in Jordan: assessing knowledge and attitudes among dentists

**DOI:** 10.3389/froh.2025.1619119

**Published:** 2025-08-08

**Authors:** Tamadur Mahmoud Falah, Sabha Mahmoud Alshatrat, Jumana M. Sabarini, Majd M. Alsaleh, Yousef Saleh Khader, Alaa Fawwaz Dalky, Bayan Jamal Almahasneh, Abedelmalek Kalefh Tabnjh

**Affiliations:** ^1^ Department of Applied Dental Sciences, College of Applied Medical Sciences, Jordan University of Science and Technology, Irbid, Jordan; ^2^ Consultant of Pediatric Dentistry, Arabella Dental Center, Irbid, Jordan; ^3^ Department of Pediatric Dentistry, University of Illinois Chicago, Chicago, IL, United States; ^4^ Department of Public Health, Community Medicine, Jordan University of Science and Technology, Irbid, Jordan; ^5^ Department of Health Management and Policy, Faculty of Medicine, Jordan University of Science and Technology, Irbid, Jordan; ^6^ Department of Cariology, Institute of Odontology, The Sahlgrenska Academy, University of Gothenburg, Gothenburg, Sweden; ^7^ Dental Research Unit, Center for Global Health Research, Saveetha Medical College and Hospital, Saveetha Institute of Medical and Technical Sciences, Chennai, India

**Keywords:** teledentistry, dentist, Jordan, knowledge, oral health, attitude

## Abstract

**Aim:**

to evaluate the perceptions, knowledge, and attitudes of Jordanian dentists towards teledentistry.

**Methods:**

A web-based, anonymous, self-administered closed-end questionnaire was distributed across Jordan. Conducted with a sample of 250 dentists selected through convenience sampling, the research assessed knowledge and attitudes using a pre-designed and validated questionnaire. An electronic link was sent to active Jordanian Dental Association (JDA) members. The survey consisted of three sections with closed questions.

**Results:**

A total of 250 dentists (Male 52%) participated in the study. The mean knowledge and attitude scores were 78.78 ± 15.27 and 76.81 ± 14.74, respectively. Dentists without postgraduate qualifications demonstrated significantly greater knowledge compared to those with postgraduate qualifications (*p* = 0.023). Over half of the respondents agreed that teledentistry is effective for diagnosing and providing treatment recommendations from a distance and can assist in monitoring patients' oral health. However, the majority disagreed with its application across all branches of dentistry. A significant number of participants believed that teledentistry could improve access to oral healthcare, reduce costs, save time for dentists, increase accessibility in rural and underserved areas, and reduce isolation among practitioners by facilitating peer contact and specialist support. *T*-test and one-way ANOVA were used to test the mean difference of the knowledge and attitude percentages for the subcategories. Chi-square tests revealed a significant association between knowledge and attitude toward teledentistry and demographic characteristics of the participants. Also, the test revealed a significant difference in respondents' workplace settings regarding their views on teledentistry's impact on monitoring patients' oral health (*p* = 0.026), its potential for integration into current dental services (*p* = 0.001), and time-saving benefits (*p* = 0.007). The results suggest participants with less than 10 years of experience were more likely to agree that teledentistry can improve access to oral health care (*p* = 0.039). Similarly, participants also more likely to agree that teledentistry has the potential to be integrated into current dental services (*p* = 0.029).

**Conclusion:**

Teledentistry has the potential to provide equitable, cost-effective, and high-quality dental care especially for individuals in remote areas with limited access to traditional dental care.

## Introduction

Teledentistry, a subspecialist field of telemedicine, utilizes digital communication technologies to deliver dental care, education, and consultation remotely (1). It can be used for various purposes, such as diagnosis, consultation, treatment of diseases, and patient education. It may also help save resources and reduce overall healthcare expenses (2). It has become a viable option to tackle inequalities in access to oral healthcare globally.

The global healthcare landscape has recently undergone significant transformations due to advancements in telecommunications and the growing need for accessible healthcare solutions (3). Jordan, a lower-middle-income-country in the Middle East, is no exception to this trend. While Jordan has made notable progress in integrating telehealth services into its healthcare sector, the adoption of teledentistry is still in its early stages (4).

The need for teledentistry in Jordan stems from several factors, including geographic disparities in access to dental services, a shortage of specialized dental practitioners in rural areas, and the increasing prevalence of oral diseases (4, 5). The significance of teledentistry in Jordan has been further emphasized by recent global events, such as the COVID-19 pandemic. Lockdowns, mobility restrictions, and heightened concerns about infection transmission highlighted the necessity of integrating remote healthcare options to ensure continuity of care. The COVID-19 pandemic has highlighted the significance of sustainable remote healthcare solutions. Stakeholders are increasingly acknowledging the benefits of teledentistry in enhancing access to care and reducing healthcare disparities (6). It is recommended that government-led teledentistry programs be developed to extend care to underserved populations, especially in rural communities and refugee camps (7).

In Jordan, there is a lack of comprehensive understanding regarding teledentistry among dentists. The current study aims to evaluate the perceptions, knowledge, and attitudes of Jordanian dentists towards teledentistry. By analyzing the views of dental professionals, the research aims to identify how teledentistry can improve oral healthcare delivery and enhance access to quality services across the country. The study will also provide recommendations for integrating teledentistry into Jordan's oral healthcare system to enhance equity in dental care.

## Materials and methods

This study employed a quantitative approach with a descriptive cross-sectional design. A total of 280 dentists were invited to participate, and 250 completed the questionnaire, resulting in a response rate of 89.3%. Participants were selected through convenience sampling. The required sample size was calculated as 200 using the Sample Size Calculator by Wan Nor Arifin, which is licensed under a Creative Commons Attribution-Non-Commercial-Share Alike 4.0 International License.

### Research tool

A pre-designed validated questionnaire served as the research tool to assess dentists' knowledge and attitudes regarding teledentistry (8). To ensure contextual relevance, the original questionnaire was reviewed and slightly modified. Content validity was established through expert evaluation by a panel of three senior dental academics and piloted the survey on a group of Jordanian dentist. Minor adjustments were made for clarity based on their feedback. A forward and backward translation process was used to translate the original English questionnaire into Arabic, the primary language in Jordan to ensure clarity and comprehension for all participants. Data were collected using a questionnaire, accompanied by a cover letter, which was distributed electronically through Google Forms. An electronic link was sent to active Jordanian Dental Association (JDA) members. Participants were informed that their involvement was voluntary, that they could withdraw from the study at any time, and that their responses would be processed anonymously and kept confidential. Participants were briefly informed of the study objectives at the beginning of the questionnaire. The survey was conducted in English and Arabic languages.

The survey consisted of three sections with closed questions. The first section gathered demographic information, including age, gender, workplace, qualifications, years of experience, preferred screen type (computer, projector, tablet), and average time spent using the Internet for health-related purposes. The second section consisted of eight questions assessing participants’ knowledge of teledentistry, while the third section included twelve questions assessing participants' attitudes toward the effectiveness of teledentistry.

Each item was coded from 1 to 3 (Disagree = 1, Neutral = 2 and Agree = 3). Higher scores indicate higher knowledge and a more positive attitude regarding teledentistry. A total score was calculated for each subject by summing the responses [maximum score for knowledge (8 × 3 = 24) and attitude (12 × 3 = 36)]. To standardize the scores, percentages for knowledge and attitude were calculated by dividing the individual score by the maximum possible score and multiplying by 100% [for knowledge = (individual score/24 × 100%). and attitude = (individual score/36 × 100%)]. Mean knowledge and attitude percentage, standard deviation, and frequency distribution were calculated. Data were analyzed using SPSS statistical software (Version 29). Descriptive statistics, such as percentages, were used to summarize demographic information. The mean and standard deviation (SD) of the knowledge and attitude scores were calculated. *T*-test and one-way ANOVA were used to test the mean difference of the knowledge and attitude percentages for the subcategories. Chi-square tests were conducted after categorizing the scores as low (less than 50%) or high (50% or more) to determine if there were any associations between knowledge and attitude scores and sociodemographic variables. A statistical significance threshold was set at a *p*-value ≤0.05.

Our research was conducted in full accordance with the World Medical Association Declaration of Helsinki. The study protocol was approved by the Jordan University of Science and Technology (JUST) Institutional Review Board (Reference: 2023/9).

## Results

**Figure 1:**
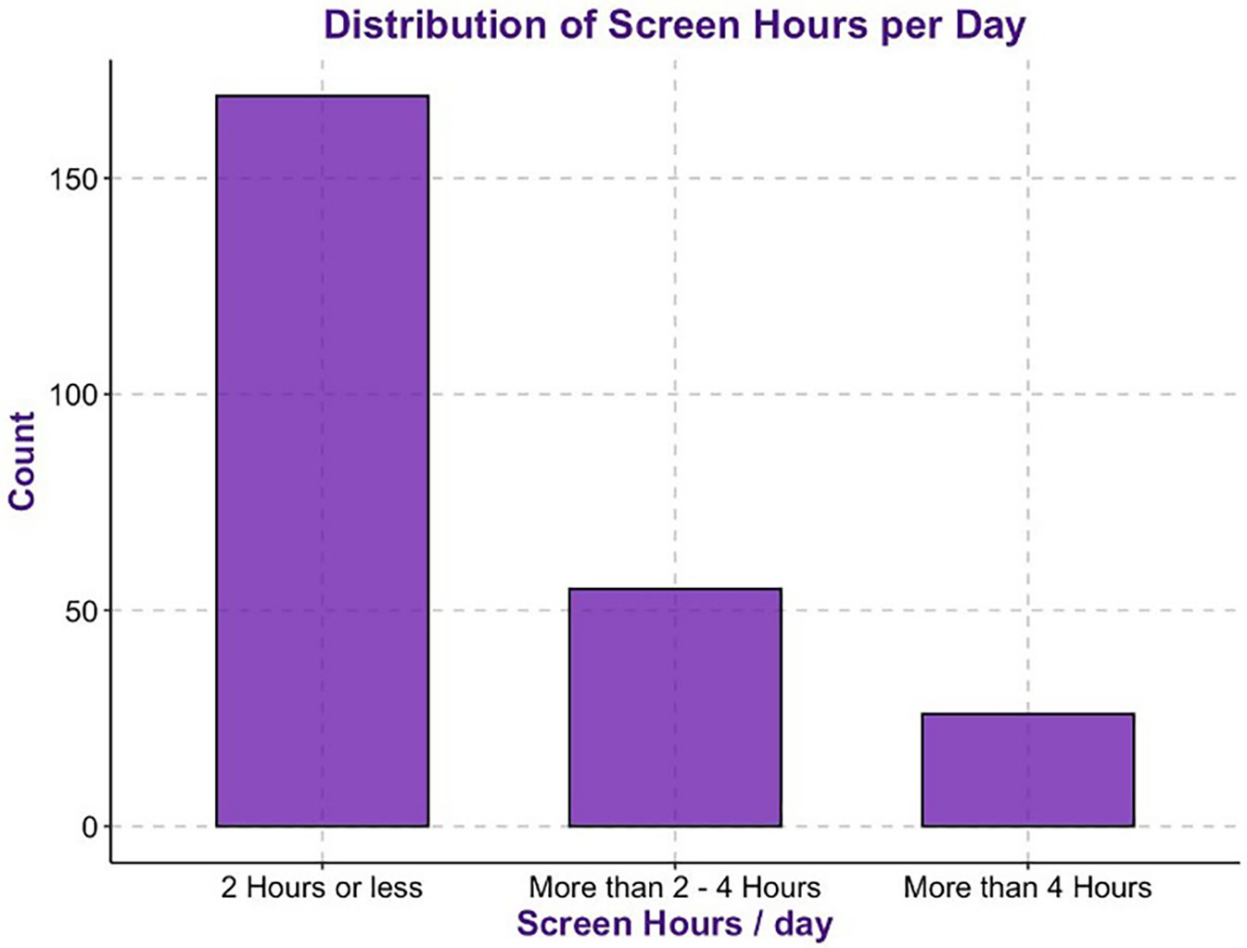
Screen and internet use for health -related purposes (average time/day).

**Figure 2:**
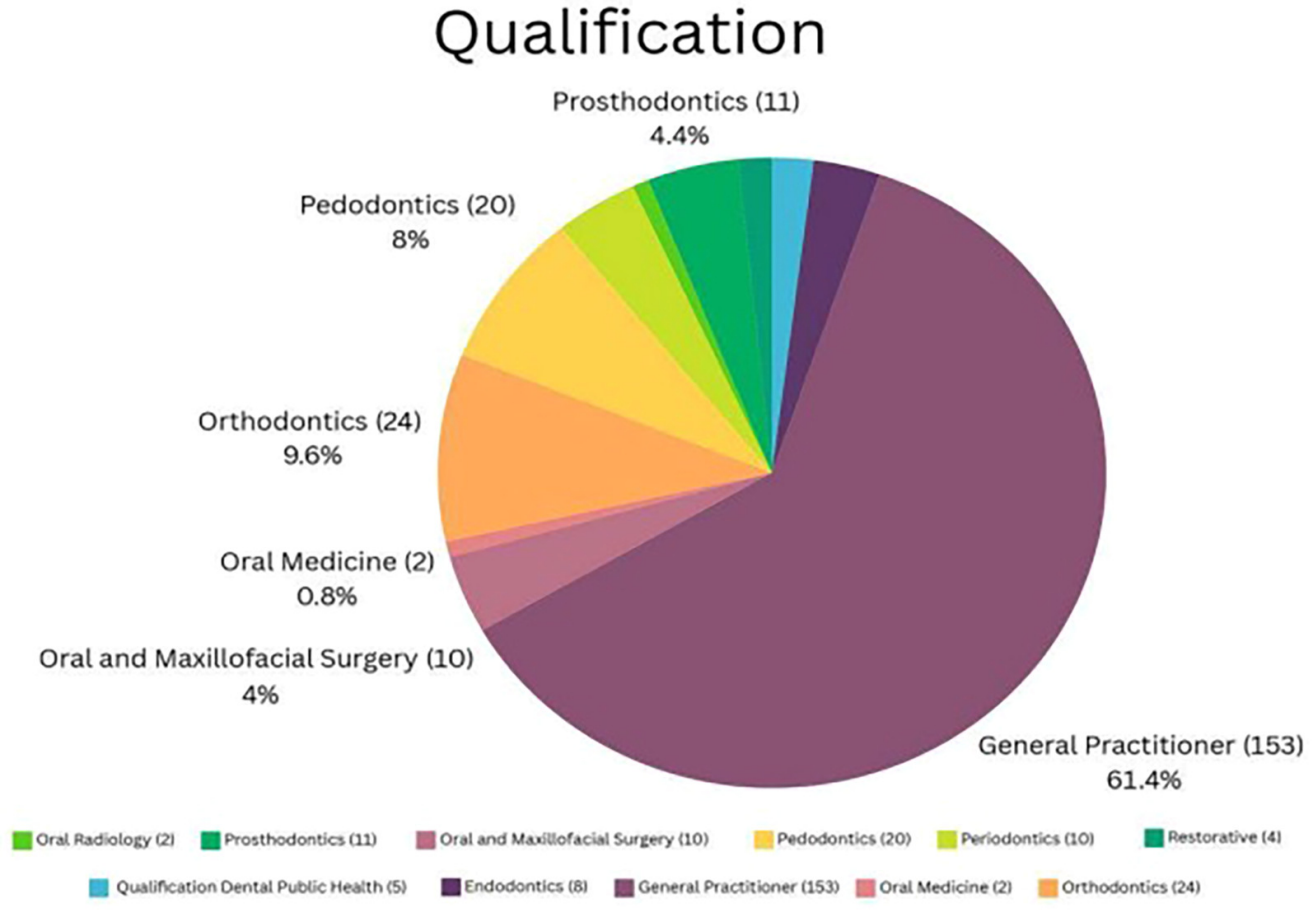
Qualification of the participants.

A total of 250 dentists from different cities in Jordan were participated in this study. Table 1 shows demographic characteristics of the participants. Regarding the time of dentists spent on screen and the internet for health-related purposes see Figure 1. Regarding the qualifications of the participants see Figure 2.

**Figure 3:**
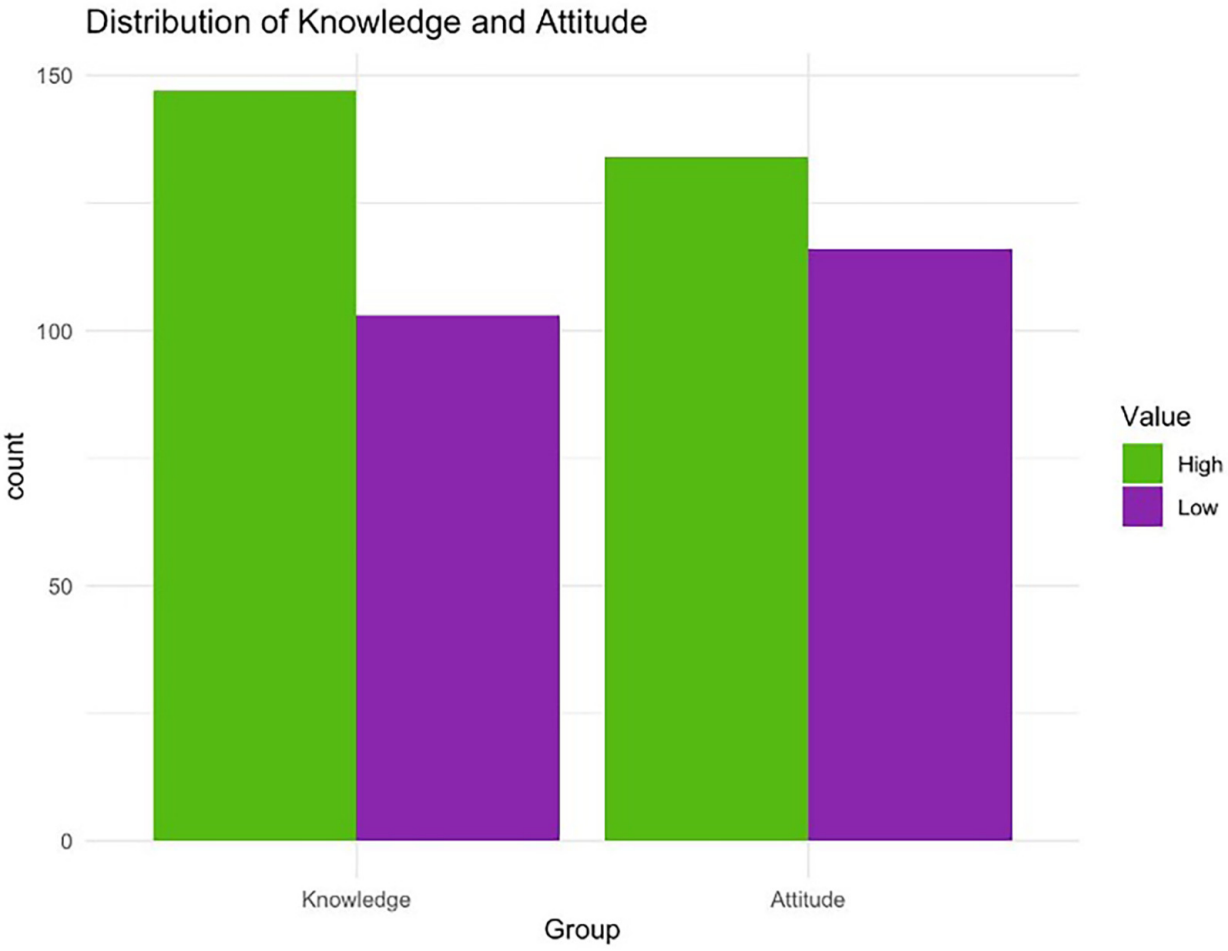
Knowledge and attitude scores as low (less than 50%) or high (50% or more).

**Table 1:** Demographic characteristics of the participants (*n* = 250).

Category		*n*	%
Gender	Female	119	47.6
	Male	131	52.4
Work place	Academic institution	27	10.8
	Ministry of health	59	23.6
	Private sector	147	58.8
	Royal medical services	16	6.4
	UNRWA	1	0.4
Years of experience	Less than 1 year	44	17.6
	(1–5) years	69	27.6
	(6–10) years	33	13.2
	(11–15) years	36	14.4
	(16–20) years	18	7.2
	More than 20 years	50	20.0

Regarding the results of the questions assessing the participants' knowledge of teledentistry, the findings revealed diverse opinions on its use. Over half of the respondents agreed that teledentistry is effective for diagnosing and providing treatment recommendations from a distance. Concerning the nature of interaction, 51.2% agreed that teledentistry does not constitute a face-to-face consultation, a significant majority (71.6%) believe that teledentistry makes it easier to consult with specialists regarding specific patient issues. In terms of education and training, the majority (56.8%) agreed that teledentistry is beneficial for dental education and training primary care dentists. Half of the respondents agreed that teledentistry can assist in monitoring patients' oral health. However, when considering its application across all branches of dentistry, the marginal majority (50.4%) disagreed. A notable number of respondents (66%) agreed that teledentistry can improve access to oral healthcare. The potential integration of teledentistry into current dental services was positively viewed by the majority (59.2%).

In the results of the survey evaluating participants' attitudes toward teledentistry, over one-third of the respondents agreed that teledentistry effectively enhances understanding of patients' oral health issues through online consultations and facilitates efficient monitoring of patients. A significant number (46.8%) of participants supported the notion that teledentistry helps lower costs. Additionally, it has the potential to save time for dentists, increase accessibility for rural and underserved communities, and reduce feelings of isolation among practitioners by facilitating contact with peers and providing specialist support, according to most participants (69.2%). Finally, when discussing the challenges of implementing teledentistry in Jordan, the majority (62%) identified poverty and inadequate infrastructure as major barriers. As detailed in Table 2. The mean knowledge and attitude scores of the study population were evident as 78.78 ± 15.27 and 76.81 ± 14.74, respectively. Dentists without postgraduate qualifications had significantly greater knowledge than dentists with postgraduate qualifications (*p* = 0.023). See Table 3 for details.

**Table 2:** Percentage distribution of responses regarding knowledge and attitudetowards teledentistry.

Knowledge	Agree *n* (%)	Disagree *n* (%)	Neutral *n* (%)
1. Teledentistry is the practice of use of Screen (computer, data show, tablet, etc.), internet, and camera technologies to diagnose and provide advice about treatment over a distance	57.6 (144)	13.2 (33)	29.2 (73)
2. Teledentistry is not a face-to-face interview.	51.2 (128)	18.4 (46)	30.4 (76)
3. Teledentistry will help to consult with an expert about a specific patient's problem	71.6 (179)	6.8 (17)	21.6 (54)
4. Teledentistry is good for dental education over the internet and for training primary care dentists	56.8 (142)	18.4 (46)	24.8 (62)
5. Teledentistry can help to monitor my patient's oral health	50.0 (125)	14.0 (35)	36.0 (90)
6. Teledentistry can be applied in every branch of dentistry	18.8 (47)	50.4 (126)	30.8 (77)
7. Teledentistry can be useful in improving access to oral health care	66.0 (165)	7.2 (18)	26.8 (67)
8. Teledentistry has potential to be integrated into our current dental services	59.2 (148)	10.8 (27)	30.0 (75)
Attitudes			
1. Teledentistry can provide a good understanding of the patient's oral health problem over the internet	44.4 (111)	17.2 (43)	38.4 (96)
2. Using teledentistry, I will be able to monitor my patient's condition well	42.4 (106)	18.0 (45)	39.6 (99)
3. Dental examinations are as accurate via screen and camera as in a traditional office setting	24.8 (62)	41.6 (104)	33.6 (84)
4. Children and parents would be receptive to having a dental examination done via screen and camera	35.6 (89)	21.2 (53)	43.2 (108)
5. Teledentistry is a convenient form of oral health care delivery that makes dental examination easier	35.6 (89)	26.4 (66)	38.0 (95)
6. Teledentistry will be a standard way of oral health care delivery	29.6 (74)	27.2 (68)	43.2 (108)
7. Teledentistry can be an addition to the regular care we (dentists) provide	64.4 (161)	6.8 (17)	28.8 (72)
8. Teledentistry can reduce costs for dental practices	46.8 (117)	20.8 (52)	32.4 (81)
9. Teledentistry can save time for me	56.4 (141)	10.8 (27)	32.8 (82)
10. Teledentistry can increase accessibility of specialists to rural and underserved communities	67.6 (169)	8.8 (22)	23.6 (59)
11. Teledentistry can reduce isolation of practitioners by providing peer contact and specialist support	69.2 (173)	6.4 (16)	24.4 (61)
12. Major challenges in teledentistry in Jordan are population below the poverty line and lack of infrastructure	62.0 (155)	8.4 (21)	29.6 (74)

**Table 3:** Assessment of mean knowledge and attitude percentage scores with demographic characteristics of dentists (*N* = 250).

Categories	Knowledge %			Attitude %	
	*n*	Mean ± SD	*p* value	Mean ± SD	*p* value
Age (in years)					
30 years or less	112	80.29 ± 12.88	0.464	78.35 ± 13.20	0.232
31–40 years	57	78.36 ± 16.95		77.39 ± 14.94	
41–50 years	36	78.12 ± 17.32		75.69 ± 14.72	
More than 50 years	45	76.11 ± 16.75		73.15 ± 17.66	
Sex					
Male	131	77.59 ± 16.02	0.201	76.80 ± 15.02	0.992
Female	119	80.08 ± 14.35		76.82 ± 14.49	
Workplace					
Academic	27	82.25 ± 18.37	0.11	80.86 ± 13.87	0.073
MIH/UNRWA	60	81.88 ± 13.63		79.68 ± 14.16	
RMS	16	78.13 ± 12.03		72.92 ± 14.18	
PS	147	76.94 ± 15.42		73.32 ± 14.97	
Qualification					
Dentists without postgraduation	157	79.06 ± 14.76	0.707	78.43 ± 14.85	0.023*
Dentists with postgraduation	92	78.31 ± 16.16		74.03 ± 14.19	
Work experience (in years)					
<6 years	113	82.02 ± 13.23	0.476	78.91 ± 12.90	0.122
6–10 years	33	78.66 ± 13.77		75.51 ± 15.17	
>10 years	104	77.48 ± 17.62		74.95 ± 16.23	
Internet access (in hours)					
2 h or less	169	78.40 ± 15.65	0.729	76.08 ± 14.67	0.53
More than 2–4 h	55	80.23 ± 13.96		78.43 ± 14.33	
More than 4 h	26	78.21 ± 15.74		78.10 ± 16.17	
Total	250	78.78 ± 15.27		76.81 ± 14.74	

Statistical tests applied: *t*-test, one-way ANOVA.

* Indicates statistically significant difference at *p* ≤ 0.05.

Figure 3 shows the knowledge and attitude scores after categorizing the scores as low (less than 50%) or high (50% or more). Furthermore, the results of Chi-square test showed a statistically significant association between knowledge and attitude toward teledentistry and demographic characteristics of the participants. The results showed a statistically significant difference in respondents' age groups regarding various perceptions of teledentistry. Specifically, participants were asked whether they believe teledentistry can improve access to oral health care (*p* = 0.018), whether it has the potential to be integrated into current dental services (*p* = 0.002), whether it can serve as an addition to regular care (*p* = 0.054), and whether it saves time (*p* = 0.029). Younger participants notably showed a more positive view on these questions, while the perception declined with increasing age.

The survey revealed a statistically significant difference in respondents' workplace settings regarding their views on teledentistry's impact on monitoring patients' oral health (*p* = 0.026), its potential for integration into current dental services (*p* = 0.001), and time-saving benefits (*p* = 0.007). These positive perceptions were notably higher among participants from academic institutions and the Ministry of Health/United Nations Relief and Works Agency (UNRWA). Additionally, participants, particularly those from the Ministry of Health/UNRWA, academic institutions, and the private sector, concurred that teledentistry enhances access to specialists for rural and underserved communities (*p* = 0.006).

The survey highlights how participant's specialty areas also play a key role in the perception of teledentistry. The result showed a statistically significant difference among respondents’ specialty area when asked if the dental examinations are accurate via Screen and camera. The results suggest Oral and maxillofacial surgery (OMS), Endodontics, and orthodontics were less likely to agree that the dental examinations through computer, tablet, and camera are accurate compared to traditional in -office settings (*p* = 0.028). Similarly, OMS, periodontics, and endodntics showed disagreement with the view that teledentistry is a convenient form of oral health care delivery which makes dental examination easier (*p* = 0.008).

The survey highlights how participants' years of experience influence their perception of teledentistry. The result showed a statistically significant difference among respondents' years of experience when asked if the teledentistry can be useful in improving the access to oral health care and teledentistry has a potential to be integrated into current dental services. The results suggest participants with less than 10 years of experience were more likely to agree that teledentistry can improve access to oral health care (*p* = 0.039). Similarly, participants also more likely to agree that teledentistry has the potential to be integrated into current dental services (*p* = 0.029).

## Discussion

This study examined the views of Jordanian dentists on teledentistry and its integration into practice. The results indicate that teledentistry can help overcome barriers to accessing oral healthcare, aligning with global trends. Studies have demonstrated that teledentistry improves accessibility, reduces travel and waiting time, and enables timely consultations, especially in rural or low-resource settings (9–11). Participants recognized its diagnostic and therapeutic value, with many agreeing it enhances access to care. This supports findings from Tiwari et al. (12) which highlighted teledentistry's role during the COVID-19 pandemic, and Soegyanto et al. (13), who demonstrated its effectiveness in improving referrals and triage in remote areas, reducing unnecessary in-person visits (12, 13).

Our findings indicate that age significantly influences attitudes toward teledentistry. Younger participants were much more likely to view teledentistry as beneficial for improving access to oral healthcare, integrating it into existing dental services, serving as a supplement to regular care, and saving time. In contrast, older participants were more skeptical of these advantages. This trend aligns with global studies showing that younger healthcare providers are more inclined to adopt digital health solutions due to their greater familiarity with technology. Research has shown that younger practitioners are more likely to perceive telehealth as beneficial and are generally more adaptable to digital platforms in clinical practice (14–16). The study also shows how years of experience affect perceptions of teledentistry. Participants with less than 10 years of experience were significantly more likely to agree that teledentistry enhances access to oral healthcare and has the potential to integrate into current dental services. While younger participants may demonstrate greater technological familiarity and openness toward teledentistry, their limited clinical exposure may restrict their ability to evaluate its diagnostic limitations fully. In contrast, older or more experienced practitioners, informed by their clinical expertise, may have a more nuanced understanding of the challenges teledentistry poses in complex diagnostic and treatment scenarios (14, 15, 17).

A substantial proportion of respondents selected “neutral” for many statements. This trend may reflect a general lack of familiarity or limited exposure to teledentistry in daily practice, particularly since its implementation in Jordan remains nascent. It also suggests potential gaps in training, experience, and confidence, highlighting the need for targeted professional education to better inform dentists about the benefits and limitations of teledentistry.

Several factors contribute to the positive perception of teledentistry among public sector practitioners. First, higher patient volumes in Ministry of Health (MOH) and UNRWA clinics, especially in areas with limited access to dental specialists, make teledentistry a valuable tool for managing large patient loads. Second, public sector dentists often treat lower-income populations with limited access to care, making remote evaluations and follow-ups through teledentistry a practical solution.

Lastly, the focus on preventive care and public health in government hospitals and community clinics encourages practitioners to see teledentistry as an innovative way to enhance oral health outreach. Overall, these factors could have shaped public sector dentists' favorable attitudes toward teledentistry, highlighting its potential to improve access to oral healthcare.

Teledentistry in Jordan shows promise but is still in its early stages, with limited research available. Previous studies, such as those conducted in 2020, have identified challenges Jordanian dentists face in utilizing teledentistry, especially in diagnosing oral infections. Pilot programs in urban areas, mainly within private practices and academic institutions, have demonstrated the feasibility of remote dental care using platforms like video conferencing, digital imaging, and electronic health records (4, 6). Moreover, during the COVID-19 lockdown, Jordanian dentists used professional WhatsApp groups for consultations on oral infections and antimicrobial prescribing, indicating an informal adoption of teledentistry (4). However, integration into public healthcare services remains limited.

A study in 2023 found that Jordanian healthcare professionals recognize the value of telehealth in reducing disparities in healthcare access, particularly for rural and refugee populations (18). Nevertheless, the study highlighted a lack of formal training programs, which may hinder widespread adoption. Our findings support this observation; younger and less experienced professionals generally accepted teledentistry, underscoring the need for structured training programs.

A significant finding from this study is the variation in attitudes among different dental specialties. Oral and maxillofacial surgeons (OMS), endodontists, and orthodontists were less likely to agree that dental examinations conducted through screens and cameras are as accurate as those performed in-person. Furthermore, specialists in OMS, periodontics, and endodontics were more likely to disagree that teledentistry is a convenient method for conducting dental examinations. These concerns align with existing literature, which indicates that dental specialists who rely heavily on hands-on clinical assessments often express skepticism toward digital alternatives such as teledentistry, citing limitationsin diagnostic accuracy and the inability to perform physical examinations (19, 20).

Variations in attitudes toward teledentistry can often be observed across dental specialties. For instance, orthodontists and oral radiologists may show greater acceptance of teledentistry due to the suitability of their specialties for image-based consultations and treatment planning. In contrast, specialties such as oral surgery or prosthodontics, which rely heavily on hands-on procedures and physical impressions, may perceive teledentistry as limited in clinical application (9, 21). Another example, restorative dentistry, which involves remote clinical consultations, presents specific challenges and opportunities for teledentistry as explored in the study by Romano et al. (22), which illustrates how restorative specialists may require tailored teledentistry approaches due to the complexity of treatment planning and clinical evaluation (22). These differences highlight the importance of tailoring teledentistry solutions and training to meet the specific needs of each specialty.

Notably, only 50.4% of participants disagreed with the statement that teledentistry can be applied in every branch of dentistry. This marginal majority suggests a lack of consensus among dentists regarding the scope of teledentistry. It may indicate a need for clearer guidelines and education on its role across various dental specialties.

Teledentistry shows great promise in preventive care, diagnostics, and post-operative follow-ups. Its use of high-quality digital imaging and real-time consultations is especially beneficial in Jordan, where some areas lack dental specialists. Additionally, 56.8% of dentists surveyed in Jordan recognized teledentistry's value in professional education and training, aligning with a study conducted in (2013), that's noted its potential for ongoing professional development (8).

Barriers to teledentistry adoption in Jordan: Teledentistry in Jordan faces several barriers to widespread adoption: (1) Infrastructure Limitations, Inconsistent internet connectivity and limited access to advanced technology, especially in rural areas, are major concerns (12). (2) Digital Literacy, Differences in digital literacy among patients and practitioners hinder effective use of teledentistry platforms. (3) Culturalresistance, only 35.6% of participants view teledentistry as convenient, reflecting hesitancy towards non-traditional care models. (4) Regulatory uncertainty, Jordan lacks comprehensive policies on patient privacy and data security in teledentistry (6, 7). Similar challenges have been observed in Pakistan, where inadequate infrastructure and institutional support are significant hindrances, as noted by study conducted in (2022) (23). Similarly, there was raised concerns about data security and regulatory frameworks in telehealth practices (24).

Future Directions and Recommendations: To effectively implement teledentistry in Jordan, the following strategies are crucial: (1) Digital Infrastructure Investment: Improve internet connectivity and access to technology in rural and underserved areas. (2) Professional Training: Provide education and workshops for dentists to enhance their skills in using teledentistry platforms. (3) Regulatory Development: Create clear guidelines on patient data privacy, ethical issues, and licensing for practitioners. (4) Government Initiatives and Partnerships: Government support is vital to successfully incorporate teledentistry into the national healthcare system (13). Learning from successful international models, like the Udaipur initiative, can help Jordan effectively adapt teledentistry to improve oral healthcare accessibility and quality (25).

## Conclusion

Overall, the results demonstrate strong support for the benefits of teledentistry, particularly in improving access to care, consulting with experts, and integrating it into dental practices. However, concerns about its universal application, accuracy, regulation, cultural acceptance, and infrastructural challenges highlight areas requiring further exploration and development. In conclusion, teledentistry can provide equitable, cost-effective, and high-quality dental care, especially for individuals in remote areas with limited access to traditional services.

### Limitation

The results of this investigation are based on a cross-sectional survey that is considered to be representative and diverse. Although the calculated sample size was achieved, several limitations remain. First, the use of convenience sampling may introduce selection bias and limit the generalizability. Second, while recall bias is unlikely—since most participants had limited or no direct experience with teledentistry—responses may have been influenced by their unfamiliarity to teledentistry, Third, the study relied on self-selection and self-reported responses, which may be influenced by personal interest in teledentistry. Fourth a large proportion of participants were under 30 years old, potentially underrepresenting views of more experienced professionals.

## Data Availability

The original contributions presented in the study are included in the article/Supplementary Material, further inquiries can be directed to the corresponding authors.
